# Unilateral Diaphragmatic Paralysis in a Patient With COVID-19 Pneumonia

**DOI:** 10.7759/cureus.19322

**Published:** 2021-11-06

**Authors:** Mubasshar Shahid, Shahbaz Ali Nasir, Osama Shahid, Shumaila A Nasir, Muhammad Waleed Khan

**Affiliations:** 1 Internal Medicine, CMH Lahore Medical College and Institute of Dentistry, Lahore, PAK; 2 Internal Medicine, University College of Medicine and Dentistry, University of Lahore Teaching Hospital, Lahore, PAK; 3 General Surgery, CMH Lahore Medical College and Institute of Dentistry, Lahore, PAK

**Keywords:** self-resolving, dyspnea, neurologic complication, covid-19 pneumonia, unilateral diaphragmatic paralysis

## Abstract

Severe acute respiratory syndrome coronavirus 2 (SARS-CoV-2) is best known for causing febrile pneumonia with lung parenchymal involvement. However, that is often not the only disease presentation, as many studies have shown that coronavirus disease 2019 (COVID-19) can present with other complications involving the cardiovascular and neurologic systems. Here, we report a case of COVID-19 pneumonia presenting with a peculiar finding of unilateral diaphragmatic paralysis. The patient presented with dyspnea requiring oxygen support via a nasal cannula. He was managed with the hospital's COVID-19 treatment protocols and clinically improved within 14 days of admission. This case helps shine some light on the neuroinvasive potential of SARS-CoV-2.

## Introduction

The diaphragm is the main muscle of respiration, and injury to this muscle can severely impair a person’s ability to oxygenate their blood [1]. The diaphragm allows expansion of the thoracic cavity during inspiration. When this function is impaired, it leads to reduced chest expansion and thus hypoventilation [1]. It is innervated by the phrenic nerve, which originates from the C3, C4, and C5 cervical nerve roots [1]. Diaphragmatic paralysis is a complication that has a multifactorial etiology, including traumatic, neurologic, infectious, inflammatory, and thoracic surgery, with the latter being the most common cause [1]. Ever since its discovery, severe acute respiratory syndrome coronavirus 2 (SARS-CoV-2) has presented with a myriad of complications, both pulmonary and non-pulmonary in nature, with the latter involving the cardiovascular and neurologic systems [2]. Studies have shown that human nervous tissue expresses angiotensin-converting enzyme-2 (ACE-2), which can potentially allow SARS-CoV-2 to invade and damage the nervous system at multiple different locations [3]. Another study also showed invasion of the muscular tissue of the diaphragm in intensive care unit (ICU) patients by SARS-CoV-2, and ACE-2 was also expressed in these tissues, which would again suggest that ACE-2 plays a vital role in viral invasion into the body [3,4]. According to one study, 1.51% (23 out of 1,527 patients) of patients have shown the incidental finding of diaphragmatic paralysis [5]. Here, we present a rare case of unilateral diaphragmatic paralysis leading to a raised left hemidiaphragm in a patient infected with SARS-CoV-2. We hope our findings will prove beneficial in understanding and managing such complications.

## Case presentation

An 80-year-old South Asian male, nonsmoker, with a past medical history of essential hypertension, type 2 diabetes mellitus that was controlled on oral medications (his hemoglobin A1c done last month was 6.8%), and ischemic heart disease (underwent percutaneous coronary intervention in 2010) presented to the emergency department with complaints of fever of 101 degrees Fahrenheit for the past two days associated with cough and progressive shortness of breath for the last one day. Fever was not associated with rigors or chills and was relieved upon taking acetaminophen. The systematic review of symptoms was unremarkable. On general physical examination, his Glasgow Coma Scale (GCS) score was 15/15, and all his vital signs were within normal limits except for his oxygen saturation (SpO2), which was 88% on room air and improved to 95% on 3 L supplemental oxygen via a nasal cannula. On respiratory system examination, bilateral inspiratory crackles were heard, which were more prominent on the left side of the chest as compared with the right, and there were no findings on physical examination pointing toward diaphragmatic paralysis. His gross neurologic examination was unremarkable along with the rest of his systemic examination.

The patient was subsequently admitted as a suspected case of coronavirus disease 2019 (COVID-19) pneumonia and advised a COVID- 19 reverse transcriptase polymerase chain reaction (RT-PCR) test along with a high-resolution computed tomography (HRCT) scan of the chest. His baseline laboratory results were also sent. On initial laboratory evaluation, his COVID-19 PCR was positive for SARS-CoV-2, and the following laboratory findings were noted as seen in Table 1.

**Table 1:** Laboratory reports of the patient on admission

Laboratory parameters	Normal ranges	Patient values
Total leukocyte count	4–10 × 10^9^/L	10.8 × 10^9^/L
Neutrophils	40%–80%	90%
Lymphocytes	20%–40%	7%
Neutrophil/lymphocyte ratio (NLR)	1–3	13
Serum procalcitonin	<0.15 ng/mL	0.24 ng/mL
Serum LDH	135–225 U/L	355 U/L
CRP (quantitative)	<6 mg/L	78.3 mg/L
D-dimers	<250 ng/mL	<250 ng/mL
Prothrombin time	13 seconds	13 seconds
International normalized ratio (INR)	1	1
Activated partial thromboplastin time	32 seconds	32
Interleukin-6	<7 pg/mL	2.18 pg/mL
Alanine aminotransferase	0–42 U/L	35 U/L
Serum creatinine	62–120 μmol/L	88 μmol/L
Serum urea	2.9–8.2 mmol/L	6.9 mmol/L

**Figure 1:**
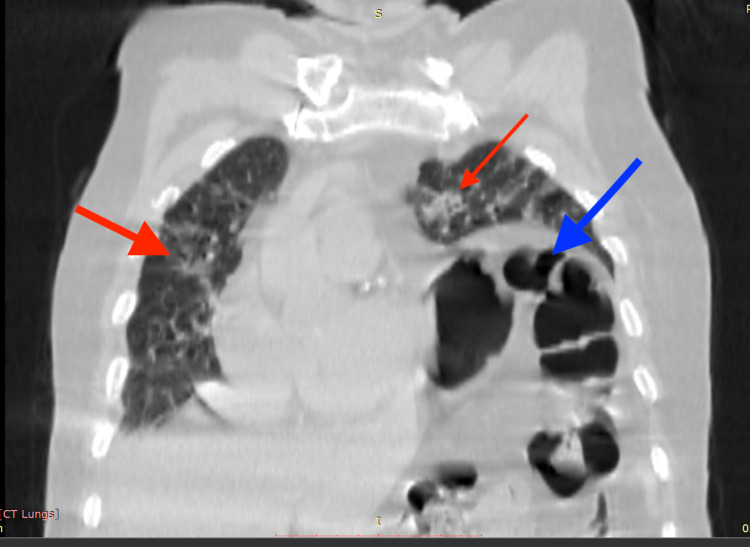
Coronal section chest HRCT showing bilateral multifocal peripheral ground glass opacities (red arrows) with a raised left hemidiaphragm showing intestinal gas bubbles in the left hemithorax (blue arrow)

**Figure 2:**
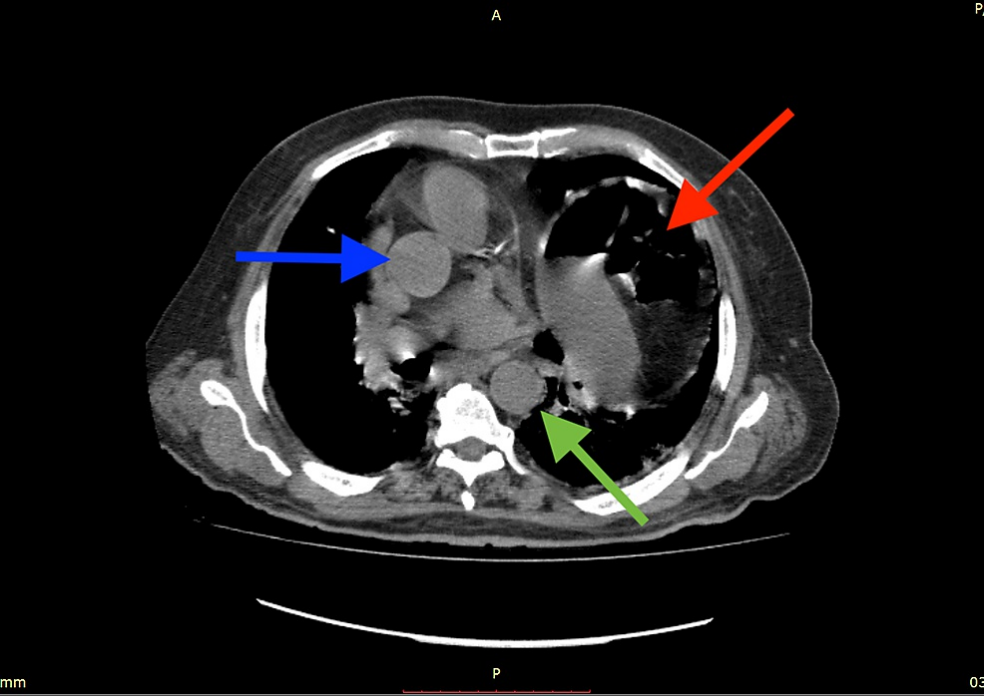
Transverse section chest HRCT showing the stomach and intestines with gas bubbles (red arrow) reaching up until the level of the arch of the aorta with ascending (blue arrow) and descending (green arrow) branches visible

**Figure 3:**
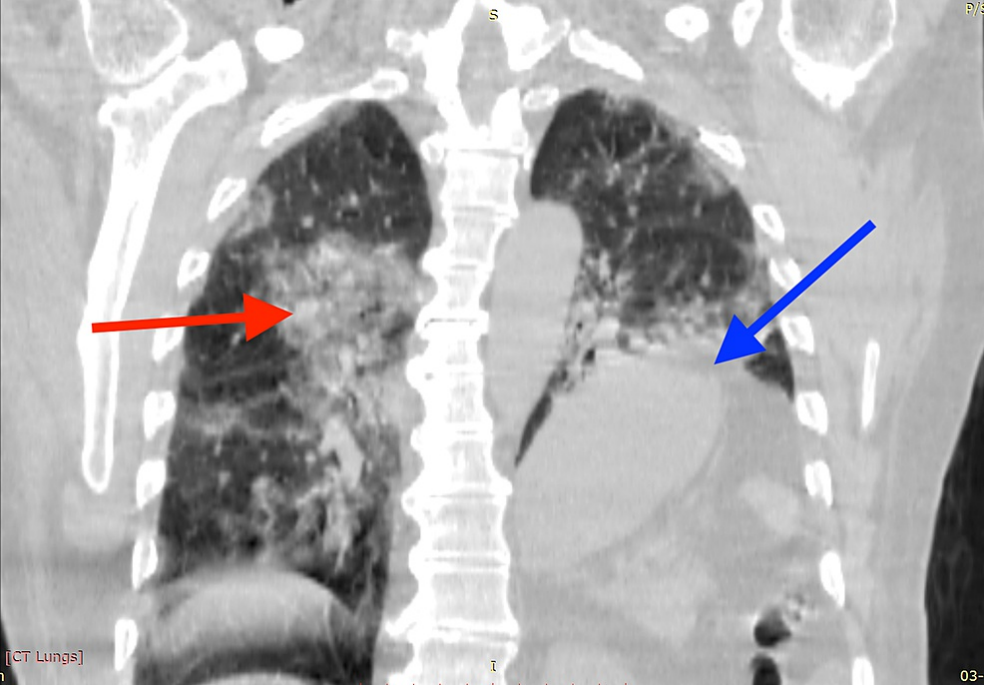
Coronal section chest HRCT showing bilateral multifocal peripheral ground glass opacities (red arrow) with a raised left hemidiaphragm (blue arrow)

His chest HRCT showed bilateral ground glass opacities with a computed tomography severity score (CTSS) of 19/40 and a coronavirus disease 2019 (COVID-19) Reporting and Data System (CO-RADS) score of 6, along with an incidental finding of elevated left hemidiaphragm with stomach gas bubbles and gut loops reaching upward until the level of the aortic arch as seen in Figures 1, 2, and 3.

COVID-19 pneumonia was diagnosed on the basis of a positive COVID-19 RT-PCR, HRCT CTSS of 19/40, and CO-RADS score of 6. The patient was shifted to the COVID medical ward, given his regular cardiac medications, and started on oral azithromycin 500 mg once daily, intravenous dexamethasone (with tight glucose monitoring) 6 mg once daily, prophylactic anticoagulation with subcutaneous low-molecular-weight heparin (LMWH) injection at 60 mg once daily, oral atorvastatin 20 mg once daily, insulin according to sliding scale, and injection remdesivir given as a 200 mg loading dose, followed by 100 mg daily for four days. The patient’s clinical condition improved after eight days with improvement in oxygen saturation to 97% on room air. He was discharged on day 14 and advised chest physiotherapy and incentive spirometry along with pulmonology clinic follow-up.

## Discussion

Each half of the diaphragm is supplied by a separate phrenic nerve (C3-C5), and involvement of only one side does not lead to significant impairment in respiratory function as the opposite diaphragm is able to compensate effectively along with the help from the external intercostal muscles [1]. In our case, unilateral diaphragmatic paralysis was seen, which is a finding that occurs in other viral illnesses such as herpes zoster and poliomyelitis, which would suggest that viral illnesses have a nonuniform disease presentation [6,7]. However, one study has reported bilateral diaphragmatic paralysis in a patient with COVID-19 [8].

From the early days of the pandemic, reports of neurologic manifestations, such as Guillain-Barré syndrome, anosmia, and encephalitis [9-12], were common, with neurologic manifestations being reported to be occurring in more than 80% of patients affected by COVID-19 according to one study [13]. With the progression of time, many newer neurologic complications were reported, such as the involvement of the cranial nerves [14]. These studies show that SARS-CoV-2 has the propensity to attack nerves all over the body and can present with a constellation of disease presentations. There are many theories being extrapolated regarding the mechanism of nervous system injury, such as direct viral entry into the nerves [15,16]. Studies have shown that human tissues infected by SARS-CoV-2 have increased expression of ACE-2; hence, ACE-2 may play a vital role in allowing viral invasion into neuronal and other structures, such as the diaphragm musculature, and worsen respiratory compromise [3,4]. Of note, in the study conducted on diaphragm musculature in intensive care unit (ICU) patients infected with COVID-19, the researchers found that epimysial and perimysial fibrosis was more than two times higher in the diaphragms of ICU patients with COVID-19 as compared with the control ICU patients [4]. It also appears that lung parenchymal involvement is not a requirement for neurologic manifestations, as one case reported a patient who had COVID-19 without lung parenchymal involvement who subsequently developed bilateral diaphragmatic paralysis [8]. We can see from this case that the neurologic manifestations of COVID-19 are likely a consequence of direct viral invasion and disruption of the nerves; however, the immune-mediated phenomenon should not be ruled out.

A study conducted by Abdeldayem et al. showed that of 1,527 patients affected by COVID-19, 23 had developed unilateral diaphragmatic paralysis, and of those 23 affected patients, 21 went on to make a full recovery of the associated diaphragmatic paralysis [5]. These findings would suggest that the mechanism of injury to the nervous structures is a reversible phenomenon similar to Bell’s palsy [17]. However, not all cases self-resolve, and long-term diaphragmatic dysfunction can occur, which may require surgical intervention such as plication [18]. Multiple factors, such as type 2 diabetes mellitus, may have played a role in causing diaphragm dysfunction, as unilateral diaphragm involvement is a known neurologic complication of diabetes mellitus [19]. However, in the case presented by Maurier, the patient infected with SARS-CoV-2 did not have any significant comorbidities and yet still developed diaphragmatic paralysis [8]. We were unable to locate any imaging studies that were done prior to patient admission; however, in accordance with the patient's clinical picture, along with our extensive literature review and current COVID-19 pandemic, we believe that SARS-CoV-2 was the likely etiology.

## Conclusions

In addition to the typical respiratory involvement, COVID-19 can also present with diaphragmatic involvement such as in our study. Given that SARS-CoV-2 has the potential to invade both nervous and muscle tissues, we believe that more neuromuscular complications will get documented as time progresses. It is also worth mentioning that neurologic complications can occur in patients with no lung parenchymal involvement or severe disease. With that in mind, it is important for clinicians to have diaphragmatic paralysis as a differential when dealing with suspected cases of COVID-19 with signs and symptoms of hypoxia. We recommend that daily respiratory and neurologic examinations should be conducted in inpatients admitted with COVID-19. We would also like to recommend that due to the current ongoing pandemic, physicians should order testing for SARS-CoV-2 when presented with a case of unexplained diaphragmatic paralysis. Since this pandemic is very recent, we believe that long-term follow-up studies will provide a clearer picture regarding prognosis and management in such patients.
